# Chronobiological Factors Related to Sleep and Neuropsychiatric Symptoms in Persons Living With Dementia

**DOI:** 10.1093/geroni/igab046.1690

**Published:** 2021-12-17

**Authors:** Nancy Hodgson, Fanghong Dong

## Abstract

Circadian rhythm disturbances (CRD) are commonly seen in people living with dementia. A clear understanding of the role of CRD in dementia etiology will be beneficial by exploring the exogenous factors (externally influence the duration of sleep hours, such as light/dark cycles) and endogenous factors (internal biological rhythm, such as diurnal cortisol pattern). This symposium will apply a chronobiological approach to study exogenous and endogenous factors that influence circadian rhythm and their effects on sleep and neuropsychiatric symptoms in persons living with dementia (PLWD). Four paper presentations will use secondary data analysis of data from the Healthy Patterns Clinical Trial (NCT03682185), a randomized controlled trial of a home-based activity intervention designed to improve circadian rhythm disorders in PLWD. We will first describe the circadian rhythm pattern reflected by endogenous factors (salivary cortisol), then examine salivary cortisol (endogenous) and white light intensity (exogenous) and on subjective sleep and neuropsychiatric symptoms (including depression) in PLWD, respectively. In session 1, we will present cortisol diurnal rhythm pattern in PLWD using a cross-sectional design. In session 2, we will discuss the relationship between salivary cortisol indicators and depressive symptoms. In session 3, we focus on the association between diurnal cortisol slope and neuropsychiatric symptoms using the baseline data. In session 4, we describe the association between evening white light exposure and subjective sleep. The discussant will describe how these findings build on our understanding the nature of circadian rhythm disturbance in dementia and inform future research and treatment approaches.

